# Comparison of Clinical and Esthetic Results of Different Techniques in the Treatment of Multiple Gingival Recessions

**DOI:** 10.1111/jerd.70018

**Published:** 2025-08-03

**Authors:** Fatma Uçan Yarkaç, Dilek Özkan Şen, Kevser Yildirim, Zeynep Taştan Eroğlu, Osman Babayiğit

**Affiliations:** ^1^ Department of Periodontics, Faculty of Dentistry Necmettin Erbakan University Konya Turkey

**Keywords:** connective tissue grafts, coronally positioned flap, gingival recession, minimally invasive surgical procedures

## Abstract

**Objective:**

This clinical study aimed to compare the short‐term (6‐month) outcomes of root coverage using a subepithelial connective tissue graft with either a modified coronally advanced flap or the tunnel technique in the treatment of recession type I multiple gingival recessions.

**Materials and Methods:**

A total of 29 patients (9 males, 20 females) aged 19–59 years were included, with 68 gingival recession defects (24 in the mandible and 44 in the maxilla). Participants were divided into two groups: Group 1 (*n* = 14) received root coverage using a subepithelial connective tissue graft with the modified coronally advanced flap, while Group 2 (*n* = 15) was treated using the tunnel technique. Clinical and esthetic outcomes were assessed at 1 and 6 months postoperatively.

**Results:**

At the 6‐month follow‐up, no statistically significant differences were found between the groups in plaque index, gingival index, papilla height, papilla width, clinical attachment level, or probing depth (*p* > 0.05). While recession depth significantly decreased and keratinized gingival width increased in both groups (*p* < 0.05), no intergroup differences were observed (*p* > 0.05). Mean root coverage was 54.26% ± 29.5% in Group 1 and 63% ± 36% in Group 2 (*p* > 0.05). Esthetic outcomes were evaluated using the Root Coverage Esthetic Score (RES), with mean scores of 6.41 ± 2.02 in Group 1 and 6.94 ± 2.09 in Group 2; no significant difference was observed between the groups (*p* > 0.05).

**Conclusion:**

Within the study's limitations, both surgical techniques yielded comparable clinical and esthetic outcomes at 6 months.

**Clinical Considerations:**

In this study, the clinical and esthetic outcomes of treating patients with multiple gingival recession defects using a subepithelial connective tissue graft with either the modified coronally advanced flap technique or the tunnel technique were compared. The results demonstrated that both techniques achieved successful outcomes.

**Trial Registration:**

ClinicalTrials.gov Identifier: NCT06509165

## 1 Introduction

Gingival recession (GR) is defined as the exposure of the root surface following apical migration of the gingival margin relative to the cementoenamel junction (CEJ) [1]. GR is clinically significant as it can lead to root sensitivity, esthetic concerns, and an increased risk of root caries [2].

Many surgical procedures have been reported in the literature for the successful management of GR. Currently, various periodontal plastic procedures have been reported to treat GR, including coronally advanced flaps (CAF) [3] modified coronally advanced flaps (MCAF) [4], laterally positioned flaps [5], and the tunnel technique (TUN [6]) with or without connective tissue grafting (CTG) [7].

Various surgical techniques have been described to address isolated GRs, with high predictability of root coverage [1, 8]. When multiple GRs involving adjacent teeth are present, an approach that addresses all defects simultaneously is preferred [9]. TUN and MCAF techniques have been proposed as effective methods for this purpose [9, 10].

TUN is recommended as a minimally invasive, safe, and predictable technique that preserves the intermediate papillae and can accelerate primary wound healing, causing less scarring with minimal trauma [11]. Additionally, TUN helps maintain adequate and continuous blood irrigation, ensuring optimal adaptation of the graft in the recipient area [12].

Another procedure for successful management of multiple adjacent GR defects is MCAF, as described in one study [4]. This technique uses a vestibular approach by avoiding trauma to the sulcular tissues as seen with conventional tunneling procedures. MCAF provides excellent esthetic results, is technically easy to perform, and can be used to treat multiple adjacent recessions [13]. One critical aspect of the technique is the attempt to advance pedicle flaps without vertical releasing incisions, as seen in the MCAF and the modified microsurgical tunnel technique [4, 14].

When the available data are examined, CAF + CTG and TUN + CTG show successful results in the treatment of multiple GRs [15], as confirmed by recent randomized controlled trials reporting favorable outcomes for both techniques [16, 17, 18, 19]. However, there is still limited evidence directly comparing TUN and MCAF, particularly in the treatment of multiple adjacent recession defects [16, 19]. This study was undertaken to help clarify the clinical differences between the TUN and MCAF techniques. Therefore, the aim of this clinical study was to compare the short‐term (6 months) results of root closure using CTG with MCAF and CTG with tunnel technique for the treatment of Recession Type 1 (RT1) multiple GRs.

Based on the current evidence on root coverage procedures, it was hypothesized that the MCAF with CTG (MCAF+CTG) would result in greater mean root coverage (mRC) at 6 months compared with the tunnel technique with CTG (TUN + CTG). We further hypothesized that both procedures would significantly reduce recession depth and increase keratinized tissue height relative to baseline.

## 2 Materials and Methods

### 2.1 Study Design and Ethical Considerations

The present article is reported according to the CONSORT statement for improving the quality of reports of parallel‐group randomized trials (http://www.consort‐statement.org/).

This was a parallel, randomized, single‐center, examiner‐ and patient‐blinded clinical trial. The GRs included in the study were classified as RT1 according to the criteria defined by Cairo et al. [20], which refers to GR with no interproximal clinical attachment loss. Two different treatment modalities were compared: the MCAF + CTG and the TUN + CTG.

The study included 30 patients, aged 19–59 (nine males and twenty females), with 70 GR defects (24 in the mandible and 46 in the maxilla) (Tables 1 and 2). The Necmettin Erbakan University Faculty of Dentistry Ethics Committee accepted this study (Decision No: 2022/235), and it was carried out in compliance with the 2000 revision to the 1975 Declaration of Helsinki. The investigation is listed in clinical trials. The recruitment of participants was conducted between January and December 2023.

**TABLE 1:** Flow chart.

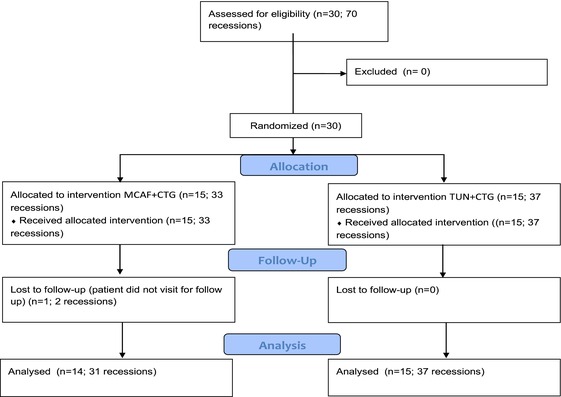

**TABLE 2:** General characteristics of the study patients (*N* = 29) and teeth (*N* = 68).

	Groups		*p*
	MCAF + CTG (Group 1)	TUNNEL + CTG (Group 2)	
Characteristics			
Age (years)	38.21 ± 10.17	38.80 ± 10.73	0.881
Sex (male/female)	3/11	6/9	0.427
Teeth *N* (%)			
Anterior	12 (35.3)	22 (64.7)	**0.023** *
Premolar	14 (48.3)	15 (51.7)	
Molar	5 (100)	(0)	
Arch, *N* (%)			
Maxilla	31 (70.5)	13 (29.5)	**< 0.001** *
Mandible	0 (0.0)	24 (100)	
Side, *N* (%)			
Right	16 (42.1)	22 (57.9)	0.625
Left	15 (50)	15 (50)	

* Bold values indicate statistical significance (*p* < 0.05, Chi‐squared test).

### 2.2 Eligibility Criteria

Patients were included in the study if they met the following criteria: age > 18 years, RT1 GR ≥ 2 mm, probing depth < 3 mm, full‐mouth plaque score < 20%, and full‐mouth gingival index score < 20%. Additionally, only patients with appropriately aligned teeth and multiple RT1 GRs located at central/lateral incisors, canines, premolars, or molars in both the maxilla and/or mandible with esthetic concerns or dentinal hypersensitivity were included. Sites with non‐carious cervical lesions (NCCLs) or non‐detectable CEJs were excluded. Patients with a history of previous periodontal plastic surgery at the affected sites were also excluded. Participants were required to be systemically healthy, non‐smokers, non‐alcoholic, and non‐pregnant. Written informed consent was obtained from all participants.

Exclusion criteria included any systemic conditions, active periodontal disease, medications (e.g., corticosteroids, immunosuppressants, bisphosphonates), or habits (e.g., smoking, alcohol use) that could interfere with healing, as well as interdental attachment loss. Patients who did not meet these criteria or declined to participate were excluded.

In our study, the initial and 6th month clinical parameters of the areas rehabilitated with connective tissue graft MCAF operation and tunnel technique in patients with RT1 GR were compared.

All patients included in the study were given detailed information about the clinical study, and their written informed consent was obtained. Individuals were divided into two groups in accordance with the study protocol.

Group 1: Patients who used connective tissue graft MCAF technique for GR closure.

Group 2: Patients who used connective tissue graft TUN for GR closure.

### 2.3 Sample Size Calculation

The sample size was calculated using *α* = 0.05, a power (1 − *β*) of 80%, and a standard deviation (SD) of 0.46 mm for reduction in recession depth, based on previous randomized controlled trials evaluating multiple adjacent GRs [15, 18, 21]. A minimal clinically important difference of 0.5 mm between groups for reduction in recession depth was adopted. According to these parameters, it was determined that 12 patients per group (MCAF + CTG and TUN + CTG) were required. Considering a possible dropout rate of 20%, the final sample size was increased to 15 Patients per group.

### 2.4 Randomization and Allocation Concealment

Participants were randomly allocated into two treatment groups using the coin toss method. A masked examiner (F.U.Y.), who was not involved in the surgical procedures or outcome assessment, performed patient enrollment and group assignment. The surgical procedures were performed by a single operator (D.Ö.Ş.), and the treatment allocation was concealed from both the participants and the outcome assessor (K.Y.).

### 2.5 Initial Therapy and Clinical Measurements

All selected patients received nonsurgical periodontal treatment, including oral hygiene instructions and supragingival scaling to ensure optimal plaque control prior to surgery. Surgical treatment of recession defects was not performed until the patient maintained adequate supragingival plaque control. Clinical measurements, including recession depth (RD) and keratinized tissue width (KTW), were performed using a periodontal probe. All measurements were conducted by a single calibrated examiner (K.Y.) at baseline and 6 months post‐surgery. The calibration process involved two repeated measurements taken 24 h apart in eight patients presenting with RT1 GR defects who were not included in the main study. The intraexaminer reliability was assessed by calculating the intraclass correlation coefficient (ICC), which showed high reproducibility (ICC = 0.92 for RD and 0.89 for KTW).

All clinical measurements were performed with a Williams periodontal probe. The plaque index and gingival index were obtained from three points: mesial, distal, and midpoints; the plaque index and gingival index were calculated according to the criteria established by Silnes and Loe [22]. GR depth (RD), papilla height (PH), papilla width (PW), probing pocket depth (PPD), clinical attachment level (CAL), and KTW were evaluated and rounded to the nearest half millimeter. The distance from the gingival margin to the base of the gingival sulcus was considered as PPD. RD was the vertical depth of the recession measured from the CEJ to the gingival margin. CAL was measured from the CEJ to the base of the gingival sulcus. KTW was measured from the mucogingival junction to the gingival margin. Gingival thickness (GT) was measured in an area 1.5 mm apical to the gingival margin with the help of an endodontic spreader having a rubber stopper. The spreader was inserted perpendicularly to the gingival surfaces until bony resistance was felt. The distance was measured with a digital caliper and rounded to the nearest 0.1 mm [23].

Percentage of root coverage was calculated as [24].

$$\frac{\mathrm{pre}\;\text{operative recession depth}-\text{post operative recession depth}}{\mathrm{pre}\;\text{operative recession depth}}\times 100$$

### 2.6 Root Coverage Esthetic Score (RES)

At the 6‐month follow‐up, esthetic outcomes were evaluated using the Root Coverage Esthetic Score (RES), a composite index designed to assess soft tissue appearance following root coverage procedures [25]. The scoring system comprises five parameters: Level of the gingival margin, scored as 0 (no root coverage), 3 (partial coverage), or 6 (complete coverage); contour of the marginal tissue, rated 0 for irregular and 1 for harmonious architecture; soft tissue consistency, where 0 indicates the presence of scarring or keloid formation, and 1 denotes normal texture; alignment of the mucogingival junction (MGJ), with 0 assigned when misaligned and 1 when properly aligned; and gingival color integration, scored 0 if the color contrasts with adjacent tissues and 1 if blending is natural. The total RES value is the sum of these individual scores, providing an overall assessment of esthetic integration post‐treatment.

### 2.7 Surgical Treatment

#### 2.7.1 Preparation of the Mcaf Receiver Area

**FIGURE 1:**
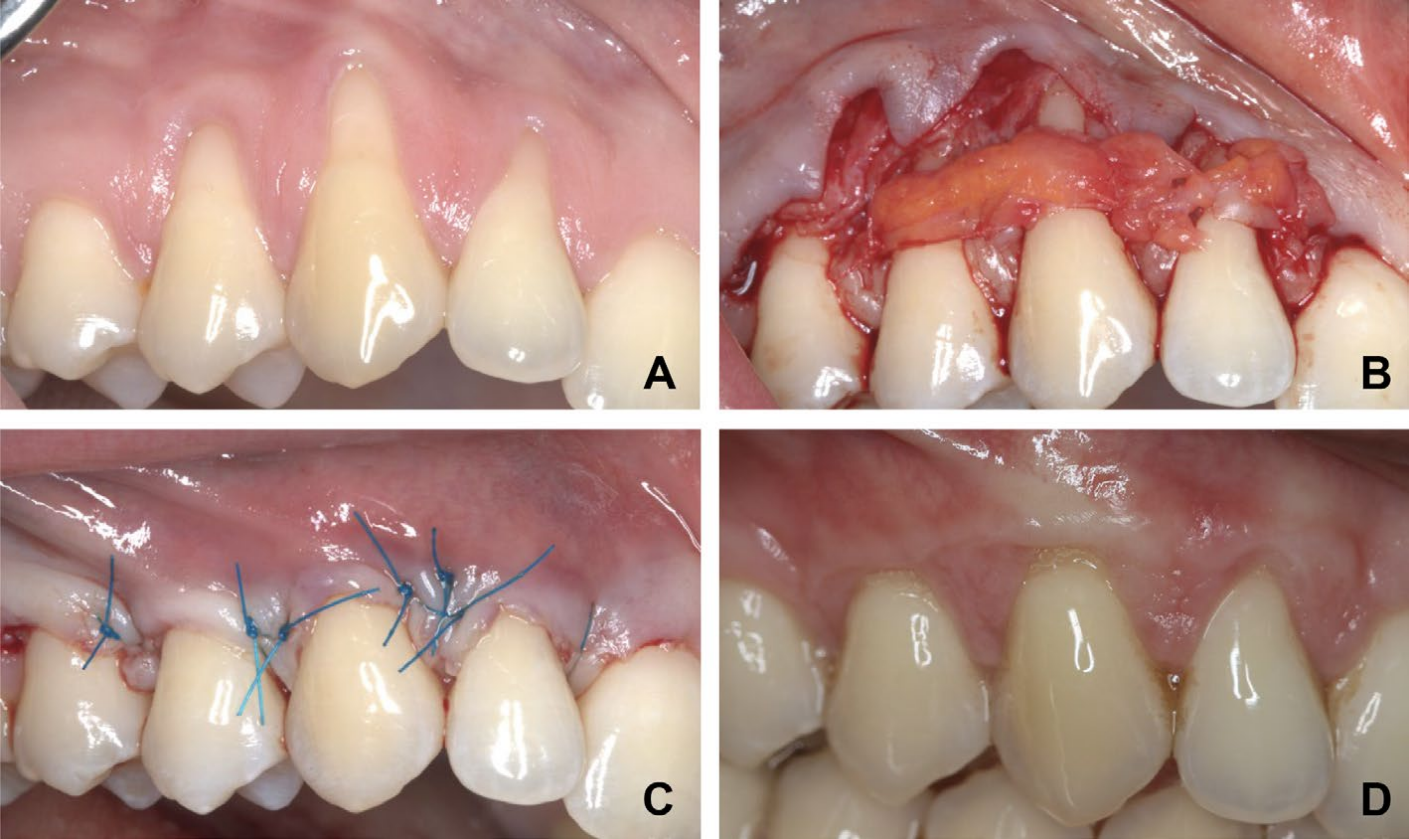
(A) Gingival recession of right lateral incisor, canine tooth, 1st premolar tooth in the upper jaw. (B) Placement of subepithelial connective tissue. (C) Coronal displacement and fixation of the flap. (D) Post operative 6th month.

Local anesthetic solution (2% lidocaine, epinephrine 1, 100.000) was injected into the areas with GR from the distant area. MCAF technique was applied as described by Zucchelli and De Sanctis [4]. All procedures were performed using microsurgical instruments (Hu‐Friedy Chicago IL, USA) and ×3.5 magnification loupes (Carl Zeiss Meditec AG, Germany). After flap removal was completed, the flap edge was positioned 1–2 mm coronal to the CEJ, and it was checked whether it remained tension‐free with cheek and lip movements (Figure 1). The root surface was flattened with Gracey curettes.

#### 2.7.2 Preparation of the Tunnel Receiver Area

**FIGURE 2:**
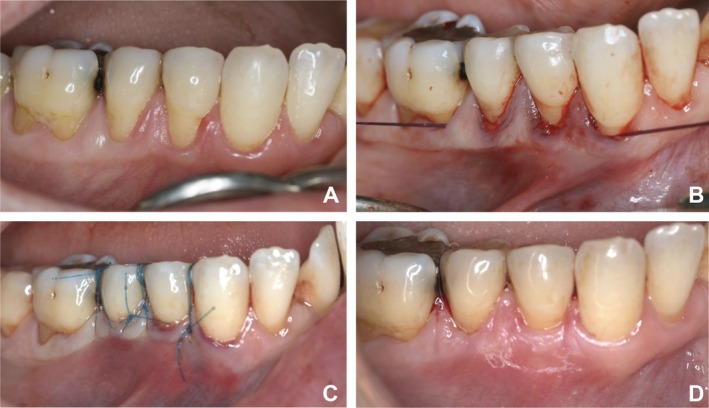
(A) Gingival recession of right canine teeth, 1st and 2nd premolar teeth in the lower jaw. (B) Placement of subepithelial connective tissue. (C) Coronal displacement and fixation of the flap. (D) Post operative 6th month.

All surgeries were performed using the TUN as described by one study [14]. After local anesthesia, the exposed root surfaces were mechanically treated with curettes (Gracey Curettes, Hu‐Friedy, Chicago, IL, USA). Tunneling knives (TKN1, Hu‐Friedy, Chicago, IL, USA) were used to prepare a split‐thickness flap and create a continuous tunnel in the buccal soft tissues, following the intrasulcular incision with a microblade (MIM64, Hu‐Friedy, Chicago, IL, USA). Split‐thickness flap preparation was performed beyond the MGJ with supraperiosteal dissection by placing the tunneling knives to the soft tissue. This process was repeated by entering through the sulcus of each tooth. After the elevation of the flap, a papilla elevator placed under the flap was entered through the sulcus to mobilize the papilla, the periosteum at the base of the papilla was cut, and the full‐thickness flap was elevated. Thus, the entire buccal soft tissue complex was mobilized coronally (Figure 2).

In both groups, CTGs were harvested from the palate using the 1.5‐mm double‐blade technique described by Harris [26], ensuring standardized thickness and minimal trauma. The grafts were trimmed and adapted to the recipient site. The CTG was stabilized by suturing it to the interdental papillae using absorbable sutures. The flap was then coronally displaced and secured with nonabsorbable sutures approximately 1–2 mm above the CEJ in both groups.

### 2.8 Postoperative Care and Controls

Cold compress was given to the patient after the operation to reduce bleeding and edema. A non‐steroidal anti‐inflammatory drug (Flurbiprofen 100 mg, Turkey) was prescribed for systemic use, to be taken three times a day for 3 days. Additionally, a mouthwash containing 0.12% chlorhexidine gluconate (Kloroben Drogsan, Turkey) was prescribed for local use, to be used twice a day for 2 weeks. After 14 days, the area was washed with physiological saline, and the healing process was clinically observed for each site. After surgery, patients were instructed to avoid any mechanical trauma and not to brush the surgical area for the first 2 weeks to allow uneventful healing. During this period, patients maintained oral hygiene by brushing the non‐operated areas and rinsing with 0.12% chlorhexidine mouthwash twice daily. After 14 days, brushing of the surgical area was initiated using the Roll technique. The patient was called for control 6 months, and clinical measurements were made using Williams sond (Hue Friedy, Chicago, USA) and control photographs were taken at baseline and after 6 months.

### 2.9 Statistical Analysis

Statistical analyses were performed with a statistical software (SPSS for Windows Version 16.0, SPSS Inc., Chicago, IL). Descriptive statistics for quantitative data were presented as means and standard deviations and as percentages for qualitative data. The Shapiro–Wilk test was used to assess the normality of continuous data. Differences between the two groups (MCAF + CTG and TUN + CTG) at baseline and 6 months were analyzed using independent samples *t*‐tests (for normally distributed data). Changes in clinical parameters (e.g., RD, PPD, CAL, KTW) from baseline to 6 months were assessed using paired *t*‐tests (for normally distributed data). Categorical variables (e.g., sex, tooth type, arch, and side) were compared using the chi‐squared test. The results were statistically significant at *p* < 0.05. However, one patient was lost to follow‐up, and their data were excluded from the analysis.

## 3 Results

One patient in Group 1, who had 2 GRs in the maxilla, did not come for follow‐up and her data were excluded from statistical analysis. Consequently, a total of 29 patients (20 women and 9 men) with a mean age of 38.52 ± 10.28 years, diagnosed with multiple GRs, were treated by a single clinician (Table 1). There were no statistically significant differences between the treatment groups in terms of age (*p* = 0.881) or sex distribution (*p* = 0.427). All the patients presented with good oral hygiene throughout the study period (FMPS < 20%; FMBS < 20%). The patients reported no postoperative complications except for having postoperative edema in all groups, which subsided in 14 days.

Treatment of 68 GRs was done and followed for 6 months. GRs in 29 patients were in 34 anterior, 29 premolar, and 5 M areas. In total, 31 areas were treated by MCAF + CTG and 37 by TUN + CTG. A statistically significant difference was seen between the two treatment procedures in relation to treated areas (*p* = 0.023) and the TUN + CTG technique was performed mostly in anterior areas (Table 2). Despite the imbalance in the distribution of treated sites, tooth localization (anterior, premolar, molar) did not have a significant effect on the clinical outcomes. The analysis revealed that the percentage of RC did not differ significantly between the treatment groups based on tooth localization (*p* = 0.138). Furthermore, when the analysis was performed according to the dental arch (mandible vs. maxilla), no statistically significant difference was observed between the two surgical approaches (*p* = 0.750). These findings suggest that although the TUN + CTG technique was more frequently applied in anterior regions, this distribution difference did not significantly influence the clinical outcomes at the 6‐month follow‐up.

### 3.1 Plaque Index

In Group 1 (MCAF + CTG), the FMPS score was 0.32 ± 0.47 (baseline). While it was 0.48 ± 0.5 at 6 months and in Group 2 (TUN + CTG), it was 0.45 ± 0.6 (baseline) became 0.59 ± 0.59 at the 6th month. This change in plaque scores was not statistically significant (*p* > 0.05) (Tables 3 and 4).

**TABLE 3:** Changes in the clinical parameters from baseline to 6 months in subepithelial connective tissue graft with the tunnel procedure.

Clinical parameters	*n*	Mean ± SD		Paired difference (mean ± SD)	*p*
		Baseline	6 months postop		
RD	37	2.62 ± 1.17	1.29 ± 0.96	1.32 ± 1.25	**< 0.001** *
KTW	37	2.91 ± 1.01	3.77 ± 1.34	−0.85 ± 1.15	**< 0.001** *
PH	37	3.25 ± 0.97	3.33 ± 1.02	−0.08 ± 0.78	0.534
PW	37	3.33 ± 1.18	3.36 ± 1.18	−0.02 ± 0.97	0.853
PPD	37	1.4 ± 0.49	1.89 ± 0.68	−0.48 ± 0.79	**< 0.001** *
PI	37	0.45 ± 0.6	0.59 ± 0.59	−0.13 ± 0.48	0.096
GI	37	0.35 ± 0.48	0.51 ± 0.5	−0.16 ± 0.37	**0.012** *
CAL	37	4.45 ± 1.44	4.87 ± 1.64	−0.41 ± 1.13	**0.041** *
GT	37	0.97 ± 0.20	1.81 ± 0.37	−0.83 ± 0.23	**< 0.001** *
%RC	37	—	63 ± 36		
RES (total)	37		6.94 ± 2.09		

Abbreviations: CAL, clinical attachment level; GI, gingival index; GT, gingival thickness %RC, percentage of root coverage; PH, papilla height; PI, plaque index; PPD, probing pocket depth; PW, papilla width; RD, recession depth; RES, root coverage esthetic score; WKT, width of keratinized tissue.

* Bold values indicate statistical significance (*p* < 0.05, paired *t*‐test).

**TABLE 4:** Changes in the clinical parameters from baseline to 6 months in subepithelial connective tissue graft with the modified coronally advanced flap procedure.

Clinical parameters	*n*	Mean ± SD		Paired difference (mean ± SD)	*p*
		Baseline	6 months postop		
RD	31	3.11 ± 1.22	1.04 ± 1.08	2.06 ± 1.48	**< 0.001**
KTW	31	2.91 ± 1.27	4.22 ± 0.84	−1.3 ± 0.98	**< 0.001** *
PH	31	3.38 ± 0.84	3.35 ± 0.91	0.03 ± 0.87	0.839
PW	31	3.24 ± 0.99	3.06 ± 1	0.17 ± 1.61	0.118
PPD	31	1.48 ± 0.5	1.64 ± 0.7	−0.16 ± 0.96	0.362
PI	31	0.32 ± 0.47	0.48 ± 0.5	−0.16 ± 0.63	0.169
GI	31	0.12 ± 0.34	0.38 ± 0.49	−0.25 ± 0.44	**0.003** *
CAL	31	4.16 ± 1.4	4.69 ± 1.61	−0.53 ± 1.25	**0.027** *
GT	31	0.95 ± 0.26	1.72 ± 0.44	−0.77 ± 0.38	**0.003** *
%RC	31	—	54.26 ± 29.5		
RES (total)	31		6.41 ± 2.02		

Abbreviations: %RC, percentage root coverage; CAL, clinical attachment level; GI, gingival index; GT, gingival thickness; PH, papilla height; PI, plaque index; PPD, probing pocket depth; PW, papilla width; RD, recession depth; REC, root coverage esthetic score; WKT, width of keratinized tissue.

* Bold values indicate statistical significance (*p* < 0.05, paired *t*‐test).

### 3.2 Gingival Index

In both the groups, a statistically significant increase in gingival index was seen from baseline to 6 months (Tables 3 and 4). However, no statistical difference between the groups was seen at the 6 months duration (*p* > 0.05) (Table 5).

**TABLE 5:** Comparison of changes in clinical parameters between groups (baseline‐6th month).

Clinical parameters	Mean ± SD (6th month)		*p*	
	MCAF + CTG (Group 1)	TUNNEL + CTG (Group 2)	Baseline	6th month
RD	**2.06 ± 1.48**	**1.32 ± 1.25**	0.097	0.321
WKT	**−1.3 ± 0.98**	**−0.85 ± 1.15**	0.999	0.107
PH	**0.03 ± 0.87**	**−0.08 ± 0.78**	0.562	0.943
PW	**0.17 ± 1.61**	**−0.02 ± 0.97**	0.722	0.266
PPD	**−0.16 ± 0.96**	**−0.48 ± 0.79**	0.523	0.151
PI	**−0.16 ± 0.63**	**−0.13 ± 0.48**	0.310	0.419
GI	**−0.25 ± 0.44**	**−0.16 ± 0.37**	**0.035** *	0.304
CAL	**−0.53 ± 1.25**	**−0.41 ± 1.13**	0.406	0.541
GT	**−0.77 ± 0.38**	**−0.83 ± 0.23**	0.710	0.398
%RC	**54.26 ± 29.5**	**63 ± 36**	—	0.285

Abbreviations: %RC, percentage of root coverage; CAL, clinical attachment level; GI, gingival index; GT, gingival thickness; PH, papilla height; PI, plaque index; PPD, probing pocket depth; PW, papilla width; RD, recession depth; WKT, width of keratinized tissue.

* Bold values indicate statistical significance (*p* < 0.05, independent *t*‐test).

### 3.3 GR Depth

In Group 2, the mean RD at baseline was 2.62 ± 1.17 mm, which was reduced to 1.29 ± 0.96 mm at 6 months. In Group 1, the mean RD at baseline was 3.11 ± 1.22 mm, which reduced to 1.04 ± 1.08 mm at 6 months. This reduction was statistically significant (*p* < 0.05) within both groups (*p* < 0.05) (Tables 3 and 4). But there was no statistically significant difference between the groups (*p* > 0.05) (Table 5).

### 3.4 Percentage Root Coverage

Group 1 showed 54.26% ± 29.5% mean percentage root coverage at 6 months duration whereas Group 2 showed 63% ± 36% at 6 months (Tables 3 and 4). There was no difference between the groups as mean root coverage percentage (*p* > 0.05) (Table 5).

### 3.5 Keratinized Tissue Width

Mean width of keratinized gingiva increased 2.91 ± 1.01 mm (baseline) to 3.77 ± 1.34 mm (6 months) in Group 2. In Group 1, it increased 2.91 ± 1.27 mm to 4.22 ± 0.84 mm. This increase was statistically significant within the group (*p* < 0.05) (Tables 3 and 4). At the end of the 6‐month period, the statistical increase in KTW between the groups was not significant (Table 5).

### 3.6 Clinical Attachment Level

In the Group 2 mean CAL was 4.45 ± 1.44 (baseline) to 4.87 ± 1.64 mm at 6 months with a mean gain of 0.42 ± 0.20 mm. In the Group 1 mean CAL was 4.16 ± 1.4 (baseline) to 4.69 ± 1.61 mm at 6 months with a mean gain of 0.53 ± 0.21 mm (Tables 3 and 4). This CAL gain from baseline to 6 months was in between the groups; no significant difference was seen (*p* > 0.05) (Table 5).

### 3.7 Probing Depth

In the Group 2 the mean probing depth was 1.4 ± 0.49 mm (baseline) to 1.89 ± 0.68 mm at 6 months. In the Group 1 the mean probing depth was 1.48 ± 0.5 mm (baseline) to 1.64 ± 0.7 mm at 6 months (Tables 3 and 4). This increase was statistically not significant between the groups (*p* > 0.05) (Table 5).

### 3.8 Gingival Thickness

In the Group 2 the mean GT was 0.97 ± 0.20 mm (baseline) to 1.81 ± 0.37 mm at 6 months. In the Group 1 the mean GT was 0.95 ± 0.26 mm (baseline) to 1.72 ± 0.44 mm at 6 months (Tables 3 and 4). This increase was not statistically significant between the groups (*p* > 0.05) (Table 5).

### 3.9 Papilla Height and Width

In Group 2, PH was 3.25 ± 0.97 mm at baseline and 3.33 ± 1.02 mm at 6 months. In Group 1, it was 3.38 ± 0.84 mm at baseline and 3.35 ± 0.91 mm at the 6‐month follow‐up (Tables 3 and 4). PW in Group 2 was 3.33 ± 1.18 mm at baseline and 3.36 ± 1.18 mm at 6 months. In Group 1, it was 3.24 ± 0.99 mm at baseline and decreased slightly to 3.06 ± 1.00 mm at 6 months (Tables 3 and 4). These changes were not statistically significant between the groups (*p* > 0.05) (Table 5).

### 3.10 Root Coverage Esthetic Score

At the 6‐month follow‐up, esthetic outcomes were evaluated using the RES, a composite index designed to assess soft tissue appearance following root coverage procedures. The mean RES was 6.41 ± 2.02 in Group 1 and 6.94 ± 2.09 in Group 2, with no statistically significant difference observed between the groups (*p* = 0.299) (Tables 3, 4, 5).

## 4 Discussion

In this study, both the MCAF and TUN techniques, when combined with CTGs, demonstrated excellent clinical outcomes for the treatment of GRs. The results showed that both techniques were successful in achieving substantial RC, with no statistically significant differences between them (*p* = 0.285). Although the TUN group exhibited a slightly higher mean percentage of root coverage (63% ± 36%) compared to the MCAF group (54.26% ± 29.5%), this difference did not reach statistical significance. This suggests that both techniques are comparable in their effectiveness for root coverage, even though the TUN group showed a trend towards a higher percentage. To the authors knowledge, there are no randomized clinical trials of MCAF and TUN (without vertical releasing incisions) for the treatment of GRs using a CTG in both groups, and so direct comparison of results with other studies was not feasible.

Currently, various surgical interventions are available for the treatment of GR [16, 27]. The management of GR has become a significant concern in periodontal surgery due to its high prevalence, particularly among patients with predisposing risk factors [28, 29, 30]. Among the established techniques, the CAF and its modified variant, the MCAF, are widely utilized, either alone or in combination with CTG [31, 32]. The MCAF technique, which includes modifications to the traditional CAF to enhance flap stability and minimize post‐surgical relapse, has been shown to provide superior root coverage outcomes. The effectiveness of MCAF has been well documented in the literature. Studies have reported high root coverage rates with MCAF, with Zucchelli and De Sanctis [33] showing a remarkable 94.6% root coverage at the 5‐year follow‐up, while another study showed an average root coverage rate of 97.2% at the 1‐year follow‐up, significantly higher than the 92.6% achieved with traditional CAF [32]. In a systematic review and meta‐analysis, the overall rate of Complete Root Coverage (CRC) achieved with the MCAF was reported to be 70%, with mean RC ranging from 51.58% to 97.27%. When comparing MCAF and CAF, both techniques demonstrated comparable outcomes in terms of CRC, RD, and CAL gain; however, MCAF was associated with a more favorable postoperative course and superior esthetic results [31]. These studies support the efficacy of MCAF, particularly in combination with CTG, in achieving excellent root coverage. Numerous studies have reported consistently positive outcomes, demonstrating the technique's high clinical success in root coverage procedures. In another study, 20 patients were treated using a modified tunnel/CTG technique. The mean root coverage from baseline to 1 year post‐surgery was 82% in the test group and 83% in the control group [34]. Ahmedbeyli et al. [35] reported superior outcomes in the MCAF + acellular dermal matrix graft group compared to CAF + acellular dermal matrix graft, including higher CRC, RD, KTW gain, and greater patient satisfaction and esthetic scores. In contrast, Skurska et al. [36] found no significant differences between MCAF + CTG and CAF + CTG groups. In our study, the MCAF group achieved a root coverage of 54.26% ± 29.5%, supporting the effectiveness of the technique. Additionally, there was a slight increase in KTW in the MCAF group (3.05 ± 0.71 mm), which aligns with the literature suggesting that MCAF provides long‐term soft tissue stability.

Another widely used approach for treating GR is the TUN technique, which can be performed as a full‐ or partial‐thickness procedure [19]. In cases where the gingival tissue is thin—common among patients with GR—a full‐thickness flap design is generally preferred, as it reduces the risk of flap perforation and enhances surgical predictability [19, 30, 34]. In the literature, randomized controlled trials combining TUN with CTG have reported MRC values ranging from 81% to 98.4% and CRC outcomes between 33.3% and 85% [34, 37, 38]. These findings demonstrate the consistent clinical performance of the TUN technique in various settings. Moreover, in a systematic review and meta‐analysis [16], the TUN technique demonstrated mean root coverage rates of 82.75% for localized defects and 87.87% for multiple recessions. The study further emphasized that TUN is particularly effective in maxillary regions and Miller Class I and II defects. Our results also align with their report, showing that the TUN technique is effective for treating both localized and multiple recessions, particularly in the maxillary region. In our study, the TUN technique group achieved a root coverage of 63%, supporting the effectiveness of the technique.

Both techniques—MCAF and TUN—have respective advantages and limitations. MCAF offers superior flap stability, improved access for dissection, and better tissue adaptation, leading to predictable outcomes. Studies showed high root coverage rates, which support MCAF's reliability in achieving long‐term root coverage [32, 33]. On the other hand, the TUN technique preserves the integrity of the interdental papillae, promotes faster healing, and enhances blood supply to the graft, yielding superior esthetic results [11]. González‐Febles et al. [19] reported that at 6 months, CRC was achieved in 80.9% of teeth treated with the TUN technique and 79.5% with CAF, with no statistically significant difference between the groups. Mean RC was also similar (TUN: 94.0%; CAF: 91.1%), indicating comparable clinical performance of both techniques. Similarly, a recent systematic review and meta‐analysis by Tavelli et al. [16], which included patients with multiple or localized GR defects, found no statistically significant difference between the TUN and CAF techniques in terms of root coverage outcomes. In a meta‐analysis, Mayta‐Tovalino et al. [39] reported that both the TUN and CAF techniques were effective in achieving root coverage and exhibited similar clinical outcomes. The mean root coverage observed in our study for the TUN group was slightly higher than the MCAF group (63% ± 36% vs. 54.26% ± 29.5%), though the difference was not statistically significant.

In the present study, both the MCAF and TUN techniques demonstrated favorable clinical outcomes for root coverage, aligning with previous literature. Although no statistically significant differences were observed in clinical parameters such as PPD and PI between groups, the GI score in the MCAF group increased significantly over time (from 0.12 ± 0.34 to 0.38 ± 0.49; *p* = 0.003). This increase may be attributed to localized inflammation during the early healing phase, possibly resulting from surgical manipulation of the papillary tissues.

While the increase in KTW was slightly greater in the MCAF group, the difference did not reach statistical significance (*p* = 0.107). Nonetheless, this finding supports the notion that MCAF may contribute to long‐term soft tissue stability, consistent with previous studies suggesting its superiority in augmenting the width of keratinized tissue. A recent systematic review also concluded that MCAF, particularly when combined with connective tissue grafts, results in higher root coverage and increased KTW compared to other surgical techniques [40]. Although the TUN technique did not significantly affect parameters such as GI or KTW, it remains a clinically effective alternative, offering the advantage of being minimally invasive. Notably, the TUN group achieved a higher mean root coverage (63% ± 36%) compared to the MCAF group (54.26% ± 29.5%); however, this difference was not statistically significant. These findings suggest that both techniques provide predictable and successful outcomes.

Despite the valuable findings of this study, several limitations should be considered. Firstly, the relatively short follow‐up period of 6 months limits the ability to assess the long‐term stability and durability of the root coverage achieved by both the MCAF and TUN techniques. Additionally, although a sample size calculation was performed, the statistical power of the study may have been insufficient to detect smaller, yet clinically relevant, differences between the groups regarding the primary outcome. Another limitation is the absence of patient‐reported outcome measures (PROMs), which could have provided further insight into the patients' perspectives. Including such measures might have revealed discrepancies between clinician and patient perceptions of esthetic outcomes. Finally, while the surgical operator was well‐trained and experienced in both techniques, the findings from a single‐center, single‐operator setup may reduce the generalizability of the results to other clinical settings with varying expertise levels. These factors highlight the need for future studies with larger sample sizes, longer follow‐up periods, and a more comprehensive approach to account for these variables in order to better understand the long‐term advantages and limitations of each technique.

## 5 Conclusion

The current study found that both surgical techniques showed successful clinical results. There are limited studies in the literature to make comparisons. Longer‐term studies are needed on the subject. In conclusion, this study demonstrates that both MCAT and TUN techniques, with the additional use of CTG, can achieve favorable clinical outcomes in the treatment of multiple GRs in the maxilla and mandibula.

## Disclosure

The authors have nothing to report.

## Conflicts of Interest

The authors declare no conflicts of interest.

## Data Availability

The data that support the findings of this study are available from the corresponding author upon reasonable request.
